# Mode of Action of the Natural Insecticide, Decaleside Involves Sodium Pump Inhibition

**DOI:** 10.1371/journal.pone.0170836

**Published:** 2017-01-26

**Authors:** Yallappa Rajashekar, Thimmappa Shivanandappa

**Affiliations:** 1 Department of Food Protectants and Infestation Control, CSIR-Central Food Technological Research Institute, Mysore, Karnataka, India; 2 Animal Bioresources Programme, Institute of Bioresources and Sustainable Development, Department of Biotechnology, Govt. of India, Imphal, Manipur, India; 3 Departments of Studies in Zoology, University of Mysore, Manasagangotri, Mysore, Karnataka, India; AgroParisTech, FRANCE

## Abstract

Decalesides are a new class of natural insecticides which are toxic to insects by contact *via* the tarsal gustatory chemosensilla. The symptoms of their toxicity to insects and the rapid knockdown effect suggest neurotoxic action, but the precise mode of action and the molecular targets for decaleside action are not known. We have presented experimental evidence for the involvement of sodium pump inhibition in the insecticidal action of decaleside in the cockroach and housefly. The knockdown effect of decaleside is concomitant with the *in vivo* inhibition of Na^+^, K^+^ -ATPase in the head and thorax. The lack of insecticidal action by experimental ablation of tarsi or blocking the tarsal sites with paraffin correlated with lack of inhibition of Na^+^- K^+^ ATPase *in vivo*. Maltotriose, a trisaccharide, partially rescued the toxic action of decaleside as well as inhibition of the enzyme, suggesting the possible involvement of gustatory sugar receptors. *In vitro* studies with crude insect enzyme preparation and purified porcine Na^+^, K^+^ -ATPase showed that decaleside competitively inhibited the enzyme involving the ATP binding site. Our study shows that the insecticidal action of decaleside via the tarsal gustatory sites is causally linked to the inhibition of sodium pump which represents a unique mode of action. The precise target(s) for decaleside in the tarsal chemosensilla and the pathway linked to inhibition of sodium pump and the insecticidal action remain to be understood.

## Introduction

In view of the environmental and ecological concerns, human health hazards, and increasing insect resistance, many insecticides have been banned or replaced by newer chemicals [1]. Mode of action of the major chemical classes of insecticides involves mainly three target sites in the nervous system: acetylcholinesterase, an enzyme of critical importance in the transmission of nerve impulse (organophosphorus and carbamates), voltage-gated sodium channels across the nerve membrane (pyrethoids and DDT), and the acetylcholine receptor (neonicotinoids) [1–5]. Selective insecticides such as juvenile hormone mimics (fenoxycarb and pyriproxyfen), ecdysone agonists and chitin synthesis inhibitors (Diflubenzuron) act on insect- specific targets that disrupt reproduction and development [6–8]. Among the insecticides derived from natural sources, azadirachtin, from the Indian neem tree, is a feeding deterrent and an insect growth regulator that suppresses fecundity, moulting, pupation and adult emergence [9–10]. Compounds that selectively act on the insect nicotinic acetylcholine receptor (neonicotinoids), such as imidacloprid, acetamiprid and thiomethaxam are among the modern insecticides used in pest management [11–13]. Avermectins, the insecticides of microbial origin, target GABA-gated chloride channels [14–15], whereas, the diamide insecticide acts on the ryanodine receptor [16–17]. Spinosyns, a new class of insecticides derived from actinomycetes, show high selectivity and low mammalian toxicity with eco-friendly behaviour [18–19].

Recent addition to the natural insecticides are decaleside I and II, novel trisaccharides isolated from the roots of *Decalepis hamiltonii* (Wight and Arn.) that are toxic to several insect species by contact with the tarsal gustatory sites but not toxic by oral or topical application [20–21]. This intriguing nature of the insecticidal action of these natural insecticides seems to involve new, unknown target(s) in insects. The insect toxicity of decaleside in the contact bioassay based on the symptoms and behaviour indicated neurotoxic nature somewhat similar to that of pyrethroids [21]. The knockdown effect and mortality and symptoms suggest that decalesides may act on neural/neuromuscular targets via gustatory chemosensilla [21–22]. In insects, the axons of the gustatory receptor neurons from the chemosensilla directly report to the thoracic-abdominal and subesophageal ganglion as in the case of *Drosophila* [23–24]. Therefore, we hypothesised that the possible mode of action of decaleside on the chemosensilla may involve interference with the neuronal transmission of nerve impulse that could lead to the knock down effect. The biochemical basis of the insecticidal action of decalesides, however, is not known at present.

Na^+^, K^+^ -ATPase, or sodium pump, is a transmembrane ion motive enzyme most important in cellular ion regulation and maintenance of membrane potential by regulating the movement of Na^+^ and K^+^ ions across the cell membrane [25–28] which is coupled to ATP hydrolysis. Na^+^, K^+^ -ATPase, a highly conserved heterodimeric protein consisting of alpha and beta subunits with a transmembrane segment and the subunits combine to give tissue specific isoforms of the enzyme [28]. It is a target for natural toxins such as cardenolides from plants and bufodienolides from plants, animals, and palytoxin from marine organisms [29–30]. The natural toxins cardenolides and bufadienolides bind to the alpha subunit interfering with the cellular functions by disrupting the cat ion exchange across the cell membrane [30]. The highly toxic palytoxin, in a unique action, binds to the N-terminal side of the alpha subunit of the sodium pump, converting it to a ion channel resulting in K^+^ efflux, Na^+^ influx and membrane depolarisation [31–35]. Recently, it has been reported that palytoxin isolated from the red alga, *Chondria armata* was extremely toxic to cockroach when injected [36] suggesting the possibility that Na+, K+ -ATPase could be a potential target for newer insecticides. A preliminary observation in our study we found *in vivo* inhibition of Na^+^, K^+^ -ATPase in decaleside-treated insects, which led to the present study wherein we present experimental evidence that Na^+^, K^+^ -ATPase (sodium pump) is involved in the insecticidal action of decaleside in insects, which leads us to postulate novel mode of action for these unique natural insecticides.

## Materials and Methods

### Chemicals

The purified enzyme, sodium potassium adenosine triphosphatase (from porcine cerebral cortex), adenosine triphosphate (ATP), bovine serum albumin (BSA) and Ouabain were purchased from Sigma chemical Co., (St. Louis MO, USA). Other chemicals were purchased from Sisco Research Laboratory Mumbai, India. Decaleside I and II were isolated and characterized (purity, 99%) from the roots of *Decalepis hamiltonii* as reported earlier [21].

### Insects

Housefly (*Musca domestica)* were reared in a mixture of sterilized bran, milk powder and water, and the adults were allowed free access to water and thick paste of condensed milk and milk powder [37]. The German cockroach (*Blatella germanica)* was reared in plastic tubes with harborages, with dry food (biscuits) and water provided *ad libitum* [38]**.** Insect cultures were maintained at 25.0 ± 2.5°C and 70% RH with a photoperiod of 12:12 (L: D).

### Insecticidal activity

Knockdown effect, defined as the state of intoxication and partial paralysis with lack of movement which usually precedes death by exposure to decaleside I and II, was investigated in contact bioassays using adults of the cockroach and housefly. A 1 ml solution (in methanol) each of the decalesides containing known concentration of the compounds was applied on Whatman No.1 (9cm) filter paper and placed in a glass Petri dish and the solvent was allowed to evaporate for 10 min under airflow with a fan, followed by the release of 10 adult cockroaches or 20 houseflies into each dish. The control filter paper discs were treated with the solvent only. Methanol as solvent was chosen for the ease of evaporation. Each treatment consisted of four replicates. The knockdown of insects was recorded after 45 min exposure. The dosages ranged from 0.004 to 0.272 mg/cm^2^, and the effective dosages were chosen based on trial experiments. Four replicates were used for each dosage. KD_50_ (50% knockdown in 24 h exposure) were determined from the dose-response data using probit regression analysis [39].

### Dose-response

Decaleside I and II (0.008–0.270 mg/cm^2^) solutions (1ml) were sprayed on to filter paper and the control groups received only the solvent as described earlier. The solvent was allowed to evaporate for 10 min followed by the release of 10 unsexed adults of *B*. *germanica* and *M*. *domestica* separately into glass petri dishes (9cm diameter) at 25.0 ± 2.5°C. Four replicates were used for each dosage. Effective dosage for 50% knockdown effect (KD_50_) (45 min, exposure) was determined from the dose-response data using probit regression analysis [36].

### Time-course

Cockroaches or houseflies (10 per replicate) were released into Petri dish containing decaleside treated filter paper at KD_50_ dose (0.07 mg/cm^2^). The number of insects knocked-down was recorded for 0–60 min exposure.

### *In vivo* inhibition of Na^+^, K^+^ -ATPase in relation to knockdown effect

Dose-response study: The KD_25_, KD_50_, and KD_90_ doses of decaleside I and II were determined by exposing the insects (cockroach and housefly) for 45 min in contact bioassay. In the case of houseflies, the head and thorax were frozen for the enzyme assay, whereas, for cockroaches, the brain (cerebral ganglia) and the coxal muscle were dissected out and used.

Time-course study: Insects were exposed to the KD_50_ dose of decalesides in the contact bioassay and removed at various exposure time intervals (15, 30, 45 and 60 min). The tissues of the insects were dissected and assayed as described above.

### Na^+^, K^+^-ATPase assay

The tissues were homogenised in 0.1M tris-HCl buffer (pH 7.4), and centrifuged at 10, 000 x g for 15 min at 4°C and the supernatant was used for the assay of Na^+^, K^+^–ATPase [40]. The reaction mixture contained NaCl (0.14M), KCl (14mM), MgCl_2_ (3mM), ethylenediamine tetra-acetic acid (EDTA, 2mM), to which the enzyme (50μl) was added with or without 1mM ouabain in a final volume of 1ml and pre-incubated at 37°C for 10 min. The reaction was started by adding 50μl of ATP (1.5mM), incubated for 30 min at 37°C and the reaction was stopped by the addition of 0.5ml of ice-cold 10% trichloroacetic acid and centrifuged at 5000 rpm for 10 min and the phosphate content (Pi) in the supernatant was estimated [41]. The enzyme ATPase hydrolyses ATP to ADP and Pi (inorganic phosphate), and the specific activity of Na+, K+-ATPase was calculated as ouabain-inhibitable activity and expressed as Pi (μg)/mg protein.

### Tarsi-mediated contact toxicity in relation to Na^+^, K^+^ -ATPase inhibition

Requirement of direct contact of decaleside with the tarsi in the legs for the insecticidal action has been experimentally demonstrated by surgical ablation of the tarsi or blocking by molten wax, as reported earlier [21]. In order to test if the tarsi-mediated insecticidal action involves sodium pump inhibition, the activity of Na+, K+-ATPase activity was investigated in relation to the knockdown effect. The experimental procedures have been described earlier [21]. Insects were treated with decaleside by direct application of 1mg aqueous solution with or without surgical ablation, and, wax treatment and the knockdown effect(0–45 min) and inhibition of Na+, K+-ATPase activity were determined.

### Effect of hydrolysis of decaleside on the insecticidal activity and Na^+^, K^+^-ATPase activity

Chemical and enzymatic hydrolysis of decaleside and its effect on insect toxicity has been described earlier [21]. In this study, effect of hydrolysis on in vivo inhibition of Na+, K+-ATPase activity in relation to the knockdown effect was investigated.

### Effect of sugars on the insecticidal action of decaleside in relation to Na^+^, K^+^-ATPase inhibition

Since some sugars interfere with the insect toxicity of decaleside, we undertook to study if it involves sodium pump inhibition. The contact bioassay procedures with or without sugars on the toxicity have been described earlier [21]. The activity of Na+, K+ -ATPase was assayed in the treated and control insects as described above.

### *In vitro* inhibition of Na^+^, K^+^ -ATPase

*In vitro* inhibition of the enzyme, Na^+^, K^+^ -ATPase by decalesides in the tissues of houseflies, cockroaches (crude preparation) and the purified enzyme (porcine cerebral cortex) was studied. The enzyme was pre incubated with decaleside I and II (10μm– 1mM) at 37°C for 30 min, followed by the addition of 50μl of ATP (1.5mM), and incubated for 30 min at 37°C. The reaction was stopped by the addition of 1ml of ice-cold 10% trichloroacetic acid and centrifuged. Phosphate content in the supernatant was estimated [41]. The enzyme activity with or without ouabain (1mM) in the reaction mixture was calculated, and the inhibition of Na^+^, K^+^ -ATPase was determined. IC_50_ were calculated by regression analysis.

Protein content was measured by the method of Lowry et al. [42] using BSA as the standard.

### Statistical analysis

The data was analysed using one-way Anova (p < 0.05) using Statplus 2007 software. The data was expressed as means ± SE. Probit analysis was used for calculating KD_50_ [39].

## Results

### *In vivo* inhibition of Na^+^, K^+^–ATPase in relation to insect toxicity

**Fig 1:**
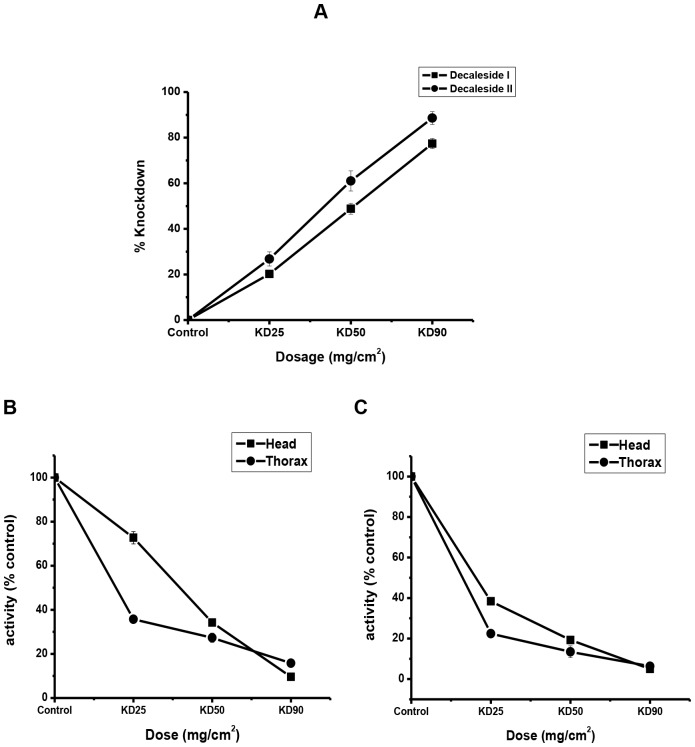
Dose-dependent *in vivo* inhibition of Na^+^, K^+^-ATPase by decalesides in relation to insecticidal activity in the house fly. **A)** % Knockdown effect (n = 4, error bars, s.e.m.). **B**) Decaleside I *(*control activity: head = 38.03 μg Pi / mg protein; thorax = 45.8 μg Pi / mg protein) (n = 4, error bars, s.e.m.).**C)** Decaleside II (control activity: head = 33.15 μg Pi / mg protein; thorax = 45.4 μg Pi / mg protein) (n = 4, error bars, s.e.m.).

Na^+^, K^+^ -ATPase activities in insects exposed to KD_25_, KD_50_ and KD_90_ doses of decaleside I and II was markedly inhibited in a dose-dependent manner in the housefly (head and thorax) (Fig 1A, 1B and 1C) and cockroach (brain and coaxial muscle) (S1A and S1B Fig). The *in vivo* enzyme inhibition was dose-dependent and correlated with the knockdown effect measured at 45 min of exposure in the contact bioassay.

**Fig 2:**
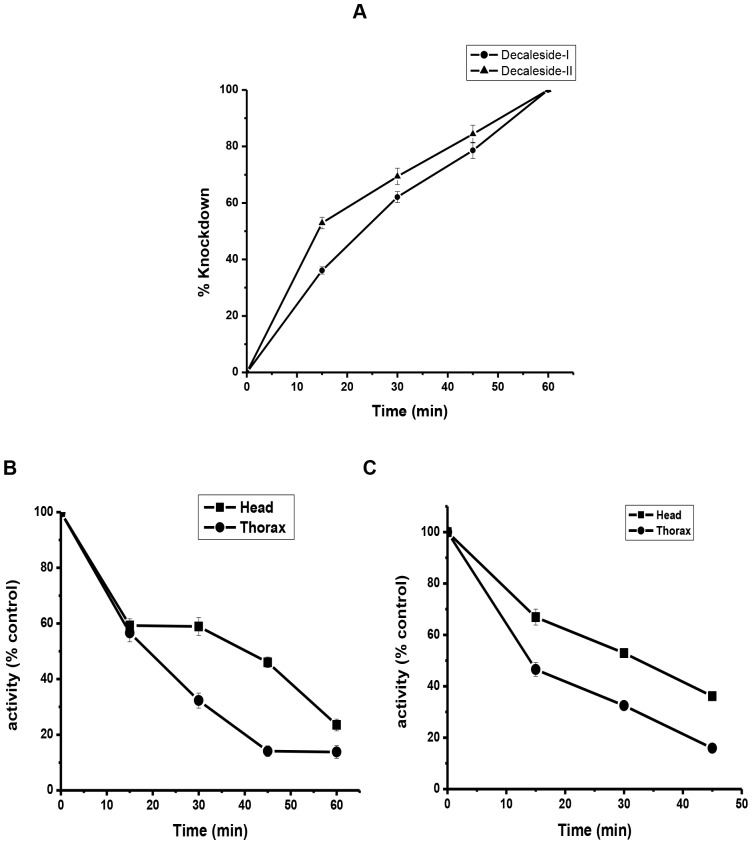
Time-course of *in vivo* inhibition of Na^+^, K^+^-ATPase in relation to knockdown of housefly treated with decalesides. **A)** % Knockdown effect (n = 4, error bars, s.e.m.). **B)** Decaleside I (control activity: head = 61.6 μg Pi / mg protein; thorax = 38.6 μg Pi / mg protein) (n = 4, error bars, s.e.m.)**,** and **C)** Decaleside II (control activity: head = 74.02 μg Pi / mg protein; thorax = 47.82 μg Pi / mg protein) (n = 4, error bars, s.e.m.).

Inhibition of Na^+^, K^+^ -ATPase markedly increased with time in insects exposed to KD_50_ dose of decalesides (Fig 2A, 2B and 2C**),** and closely correlated with the knockdown effect (S2A and S2B Fig).

Experiments in which recovery of the insects exposed to KD_50_ dose of decaleside I and II was monitored for 60–300 min after exposure, showed that recovery from knock-down effect also correlated with the recovery of the enzyme inhibition *in vivo* (S3A, S3B, S3C, S3D, S4A, S4B, S4C and S4D Figs).

### Effect of tarsal ablation and wax treatment

**Fig 3:**
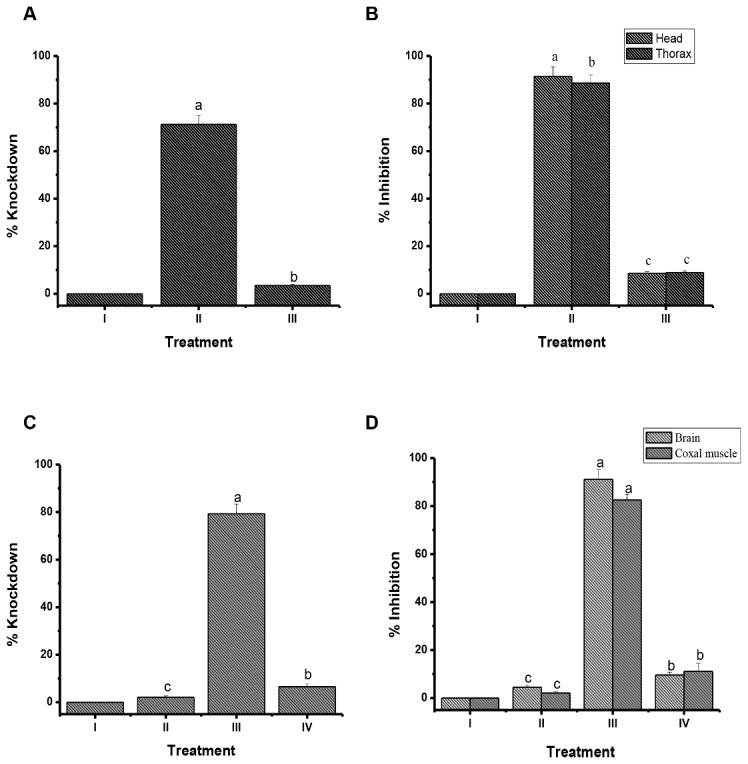
Experimental demonstration of the tarsi-mediated knockdown effect of decaleside II in relation to inhibition of Na+, K+ -ATPase activity in the brain of German cockroach. **A, B:** Effect of tarsal ablation on the **a)** Knockdown, **b)** Na^+^, K^+^ ATPase activity of decaleside II, **I)** Intact insects, **II)** Intact insects + decaleside II, **III)** Tarsii ablated + decaleside II (n = 4, error bars, s.e.m.). **C, D:** Effect of wax application on **c)** Knockdown, **d)** Na^+^, K^+^ ATPase activity in the brain of *Blatella germanica* exposed to KD_50_ (0.07 mg/cm^2^) of decaleside II, by contact bioassay. **I)** Intact insects, **II)** wax treated (solvent control), **III)** Intact insects + decaleside II, **IV)** wax treated + decaleside II. (n = 4, error bars, s.e.m.).

Both tarsal ablation and wax treatment of the tarsi in cockroaches abolished the toxic action of decaleside II as evident by lack of knockdown effect and, there was no in *vivo* inhibition of Na^+^, K^+^ -ATPase in the brain as well as coxal muscle of cockroaches (Fig 3A and 3B) or wax treatment (Fig 3C and 3D). However, in the respective control groups, toxicity correlated with Na^+^, K^+^ -ATPase inhibition.

### The effect of direct application on the tarsi

Direct application of decaleside to the tarsi of the first pair of legs induced knockdown effect and caused enzyme inhibition in the cockroaches, whereas wax application on the tarsi protected against the toxicity and, there was no enzyme inhibition (S5A and S5B Fig.**)**.

### Effect of hydrolysis

**Fig 4:**
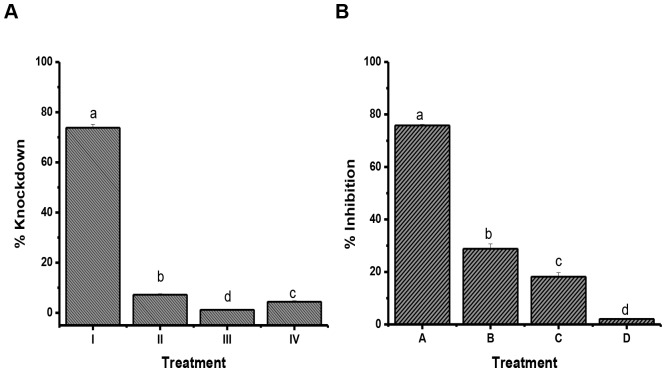
Effect of hydrolysis of decaleside II on the knockdown and inhibition of Na^+^, K^+^ ATPase activity in the brain of *Blatella germanica* exposed to KD_50_ (0.07 mg/cm^2^) of decaleside II by contact bioassay. **A)** Knockdown, **B)** Inhibition of Na^+^, K^+^ ATPase of activity. **I)** Control (without hydrolysis), **II)** Acid hydrolysis, **III)** β-Galactosidase, **IV)** α-Glucosidase. (n = 4, error bars, s.e.m.) One-way ANOVA, ***P < 0.001.

In cockroaches exposed to KD_50_ concentration of the hydrolyzed (chemical and enzymatic) decaleside II, there was no toxicity as evident from the absence of knock down. Also, there was no inhibition of Na^+^, K^+^ -ATPase in the brain of cockroaches showing correlation with lack of toxicity of the hydrolyzed sample of decaleside II (Fig 4A and 4B).

### Effect of sugars

**Fig 5:**
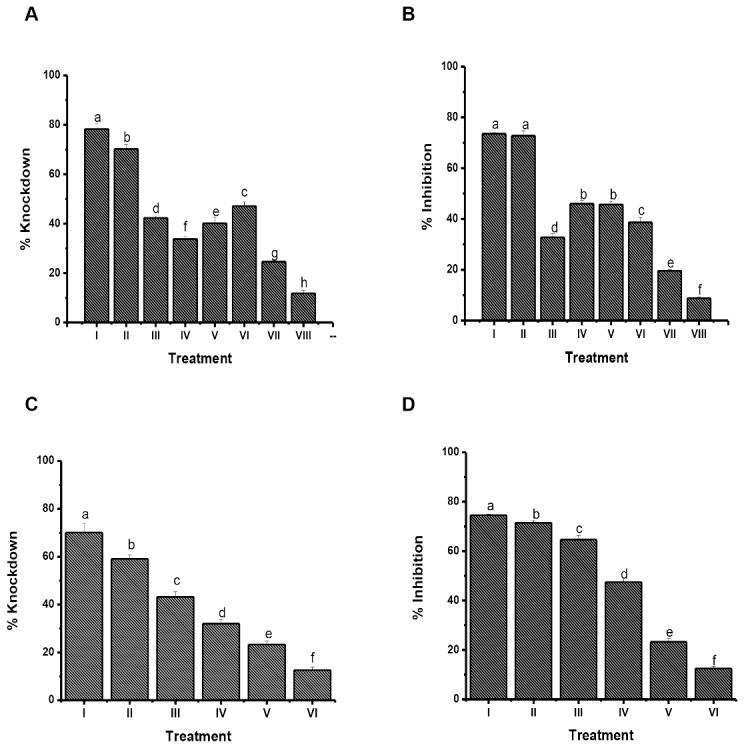
Effect of sugars on the knockdown and inhibition of Na^+^, K^+^ ATPase activity in the brain of *Blatella germanica* exposed to KD_50_ (0.07 mg/cm^2^) of decaleside II by contact bioassay. **A, B: A)** Knockdown, **B)** Inhibition of Na^+^, K^+^ ATPase: **I**. Decaleside II, **II**. Decaleside II + Glycine, **III**. Decaleside II + Glucose, **IV**. Decaleside II + Xylose, **V**. Decaleside II + Trehalose, **VI**. Decaleside II + Raffinose, **VII**. Decaleside II + Melezitose, **VIII**. decaleside II + Maltotriose in 1:1 equimolar concentration. **C, D: Effect (dose-response) of maltotriose on the C)** Knockdown, **D)** Inhibition of Na^+^, K^+^ ATPase activity: **I**. Decaleside II, **II**. Decaleside II + Maltotriose (1:0.1), **III**. Decaleside II + Maltotriose (1:0.25), **IV**. Decaleside II + Maltotriose (1:0.5), **V**. Decaleside II + Maltotriose (1:0.75), **VI**. Decaleside II + Maltotriose in 1:1 equimolar concentration.

Experiments in which cockroaches were exposed to decaleside treated paper with or without various sugars, showed that sugars interfered with the toxicity as measured by the knockdown effect, which correlated with the decreased inhibition of Na^+^, K^+^ -ATPase enzyme in the brain *in vivo* (Fig 5A and 5B**).** However, treatment with the amino acid (glycine) had no effect on the knockdown effect of decaleside II, which correlated with the lack of Na^+^, K^+^ -ATPase inhibition (Fig 5A and 5B). Among the sugars, maltotriose, a trisaccharide, was most effective in rescuing the knockdown effect of decaleside II and Na^+^, K^+^ -ATPase inhibition. The dose-dependent protective effect of maltotriose against toxicity (knock-down) of decaleside II correlated with inhibition of Na^+^, K^+^ -ATPase in the brain *in vivo* (Fig 5C and 5D).

### *In vitro* inhibition

**Fig 6:**
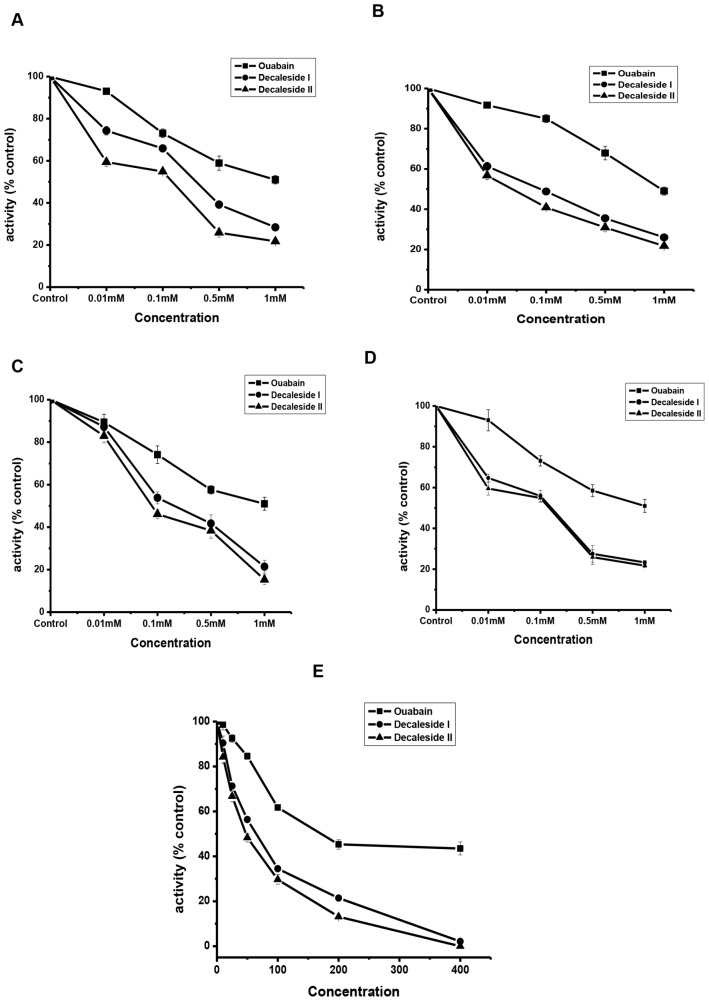
*In vitro* inhibition of Na^+^, K^+^ ATPase by decaleside I and II in comparison with that of ouabain. **A, B** : cockroach brain and thorax, respectively (control activity: head = 29.33 μg Pi / mg protein; thorax = 88.5 μg Pi / mg protein); **C, D** : house fly head and thorax, respectively (control activity: head = 73.6 μg Pi / mg protein; thorax = 97.42 μg Pi / mg protein ); **E:** Purified Na^+^, K^+^ ATPase (porcine cerebral cortex, sigma); **IC**_**50**_**:** Ouabain, 25.4 × 10^-5^M; Decaleside I, 10.5 × 10^-5^M; Decaleside II, 9.5 × 10^-5^M.

Both decaleside I and II were inhibitors of Na^+^, K^+^ -ATPase from the tissues of house fly, cockroach and the purified enzyme from the porcine cerebral cortex. The enzyme inhibition was concentration-dependant (Fig 6A, 6B, 6C, 6D and 6E). IC_50_ determined for the house fly, cockroach, and the purified Na^+^, K^+^ -ATPase indicated that decaleside I and II were more potent inhibitors of Na^+^, K^+^ -ATPase than ouabain (S1 Table).

### Type of inhibition

Kinetic studies showed that the K_m_ (ATP) shifted with the increased concentration of the inhibitors indicating that the inhibition was competitive as evident from the Lineweaver-Burk plot (S6A, S6B, S6C and S6D Fig). Similar results were found with the purified Na^+^, K^+^ -ATPase from porcine cerebral cortex, indicating the competitive type of inhibition (S7A and S7B Fig).

## Discussion

Our study has demonstrated that Na^+^, K^+^ -ATPase is severely inhibited in insects (both house fly and cockroaches) exposed to decaleside I and II in the contact bioassay. The *in vivo* inhibition closely correlated (r = >0.9) with the knockdown effect of decaleside I and II in dose-response and time-course studies. The *in vivo* inhibition was seen in both head and thorax in the case of house fly and the nervous (ganglion) and muscle tissue in cockroaches exposed to decaleside. Further, experimental evidence shows that the inhibition of Na^+^, K^+^ -ATPase requires contact of the insect leg (tarsi) with insecticide treated surface, since no inhibition was seen in insects with tarsi ablated. The same was demonstrated with the application of wax on the tarsi, wherein toxic action of decaleside was abolished. In these experiments in which contact with tarsi for the insecticidal action of decaleside is required, also show concomitant inhibition of Na^+^, K^+^ -ATPase *in vivo*. The results lead us to conclude that tarsi-mediated insect toxicity of decaleside involves Na^+^, K^+^ -ATPase inhibition. Our results are the first report of a natural insecticide (novel trisaccharides) showing Na^+^, K^+^ -ATPase as the target in its mode of action. The molecular mechanisms involved in the insecticidal action of decaleside via the gustatory chemosensilla that lead to the knock-down effect, the toxic outcome finally leading to mortality, are not clear at present. Our results show that the insects exposed to decaleside, are initially hyperactive indicating neural excitation, which is followed by knockdown effect, symptoms somewhat similar to that of pyrethoids, suggesting a neurotoxic effect [43–44]. The basic difference, however, is that the decaleside action is mediated by contact with tarsi, unlike that of pyrethoids which act by contact at any point of the body surface of insects. It is known that pyrethroids act by interfering with the voltage gated sodium channels in the neurons which causes hyper excitation leading to knockdown [45–47]. In the case of decaleside action, inhibition of sodium pump is clearly demonstrated in our studies. The following hypothesis is proposed in order to explain the mode of action of decaleside via the gustatory receptors: on contact with the gustatory (sugar) receptors (step I) causes inhibition of sodium pump (step II) which is responsible for the hyperactivity due to increased neuronal excitation caused by excessive Na^+^ concentration (step III) finally leading to knockdown effect and mortality. Electrophysiological evidence is needed to support this hypothesis.

Our study shows that some of the sugars particularly maltotriose, a trisaccharide, interferes with the insecticidal activity of decaleside implying the involvement of sugar receptors in the gustatory chemosensilla which is also concomitant with the Na+, K+ -ATPase inhibition *in vivo*. Whether specific gustatory receptor neurons mediate the action of decaleside action needs to be investigated.

Our *in vitro* studies of Na^+^, K^+^ -ATPase from the insect tissues as well as the purified enzyme indicate that the inhibition is competitive type which suggests that decaleside is interacting with the ATP binding site of the enzyme. It is known that the ATP binding site of Na^+^, K^+^ -ATPase in the plasma membrane is interior to the cell unlike the ouabain binding site which is to the exterior [25, 48–49]. The difference in the IC_50_ values for the *in vitro* inhibition of the insect enzyme versus the purified enzyme could be attributed to the crude enzyme preparation from insect tissue and it is not clear if the binding sites for decaleside on the enzyme are different. Future investigations on insecticidal action of decalesides targeting the gustatory (sugar) receptors and the neurons will open up a new field for scientific inquiry in order to unravel the mechanisms involved in their toxic action in insects. Decalesides, therefore, represent novel natural insecticidal molecules with a unique mode of action.

## Supporting Information

S1 Fig *In vivo* inhibition of Na^+^, K^+^-ATPase in the cockroach by decaleside II (contact bioassay). **A: % Knockdown. B**: Dose-dependent *in vivo* inhibition of Na^+^, K^+^-ATPase in the cockroach by Decaleside II *(*control activity: Brain = 58.03 μg Pi / mg protein; Coxal muscle = 65.8 μg Pi / mg protein).
(TIF)

S2 Fig Correlation between knockdown effect and Na^+^, K^+^ ATPase inhibition *in vivo* in house fly (A) and cockroach (B) treated with decaleside II in a dose-response study. (TIF)

S3 Fig Time-course of *in vivo* inhibition of Na^+^, K^+^-ATPase in *Blatella germanica* by decaleside II. **A) %** Knockdown, **B)** Na^+^, K^+^ ATPase activity of decaleside II in *Blatella germanica* exposure at 1mg/leg (fore legs) by topical application *(*control activity: brain = 68.03 μg Pi / mg protein; coxal muscle = 75.8 μg Pi / mg protein). **C) %** Knockdown, **D)** Na^+^, K^+^ ATPase activity of decaleside II in *Blatella germanica* exposure at KD_50_ (0.07 mg/cm^2^) by contact bioassay *(*control activity: brain = 73.4.03 μg Pi / mg protein; coxal muscle = 78.8 μg Pi / mg protein).
(TIF)

S4 Fig Time-course of *in vivo* inhibition of Na^+^, K^+^-ATPase in relation to recovery from knockdown of house flies treated with decaleside I and II at KD_50_ concentration (0.032 mg/cm^2^) (starting time point after exposure was 45 min from which recovery was monitored). **A)** Recovery from knockdown of decaleside I*;* **B)** Recovery of Na^+^, K^+^-ATPase inhibition (control activity: head = 37.56 μg Pi / mg protein; thorax = 17.4 μg Pi / mg protein). **C)** Recovery from knockdown of decaleside II*;* **D)** Recovery of Na^+^, K^+^-ATPase inhibition (control activity: head = 37.56 μg Pi / mg protein; thorax = 17.4 μg Pi / mg protein).
(TIF)

S5 Fig Effect of direct topical application of decaleside II on the tarsi with or without wax treatment on A) Knockdown, B) Na^+^, K^+^ ATPase activity in contact bioassay. **I)** Control (no wax), **II)** Control (no wax) + decaleside II (1mg/insect), **III)** wax treated on tarsi (+ solvent), **IV)** with wax treated tarsi + decaleside II (1mg/insect) (n = 4, error bars, s.e.m.), One-way ANOVA, ***P < 0.001.
(TIF)

S6 Fig Kinetics of *in vitro* inhibition of Na^+^, K^+^ ATPase in brain and coxal muscle of German cockroach: The double reciprocal (Linweaver-Burk) plot. **Brain: A,** Decaleside I**; B,** Decaleside II**. Coxal muscle: C,** Decaleside I**; D,** Decaleside II**.**
(TIF)

S7 Fig Kinetics of *in vitro* inhibition of the purified (porcine cerebral cortex) Na^+^, K^+^ ATPase: The double reciprocal (Linweaver-Burk) plot. **A)** Decaleside I**; B)** Decaleside II**.**
(TIF)

S1 Table In vitro inhibition (IC_50_) of Na^+^, K^+^ ATPase by decaleside I and II in the house fly, cockroach and purified Na^+^, K^+^ ATPase (porcine cerebral cortex). (PDF)
