# Supplementary figures and images for: Mode of Action of the Natural Insecticide, Decaleside Involves Sodium Pump Inhibition
Source: PLoS One. 2017 Jan 26;12(1):e0170836. doi: 10.1371/journal.pone.0170836 (PMC5268410; doi:10.1371/journal.pone.0170836)

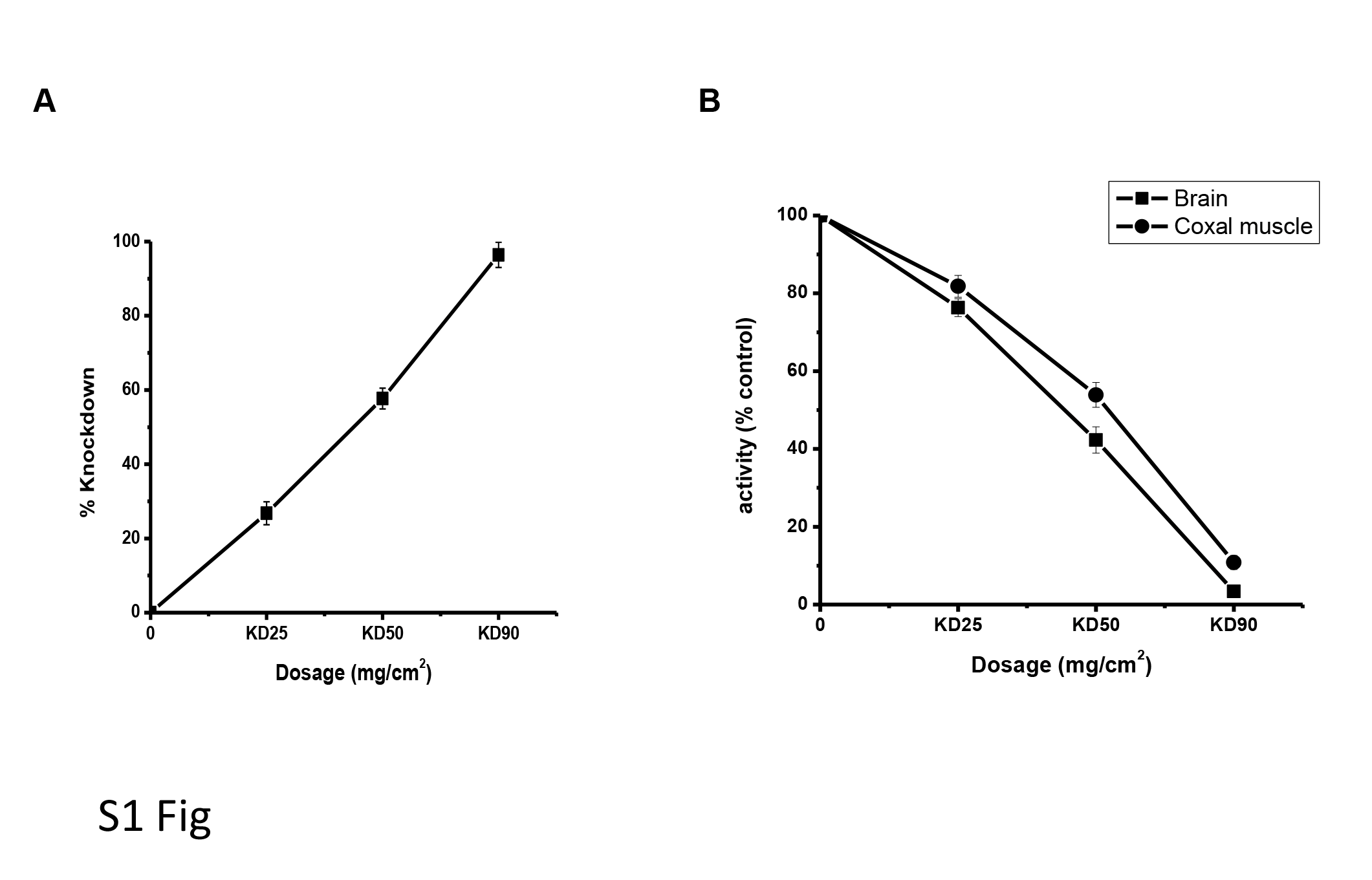

Supplement: S1 Fig — A: % Knockdown. B: Dose-dependent in vivo inhibition of Na+, K+-ATPase in the cockroach by Decaleside II (control activity: Brain = 58.03 μg Pi / mg protein; Coxal muscle = 65.8 μg Pi / mg protein). (TIF) [file pone.0170836.s001.tif]

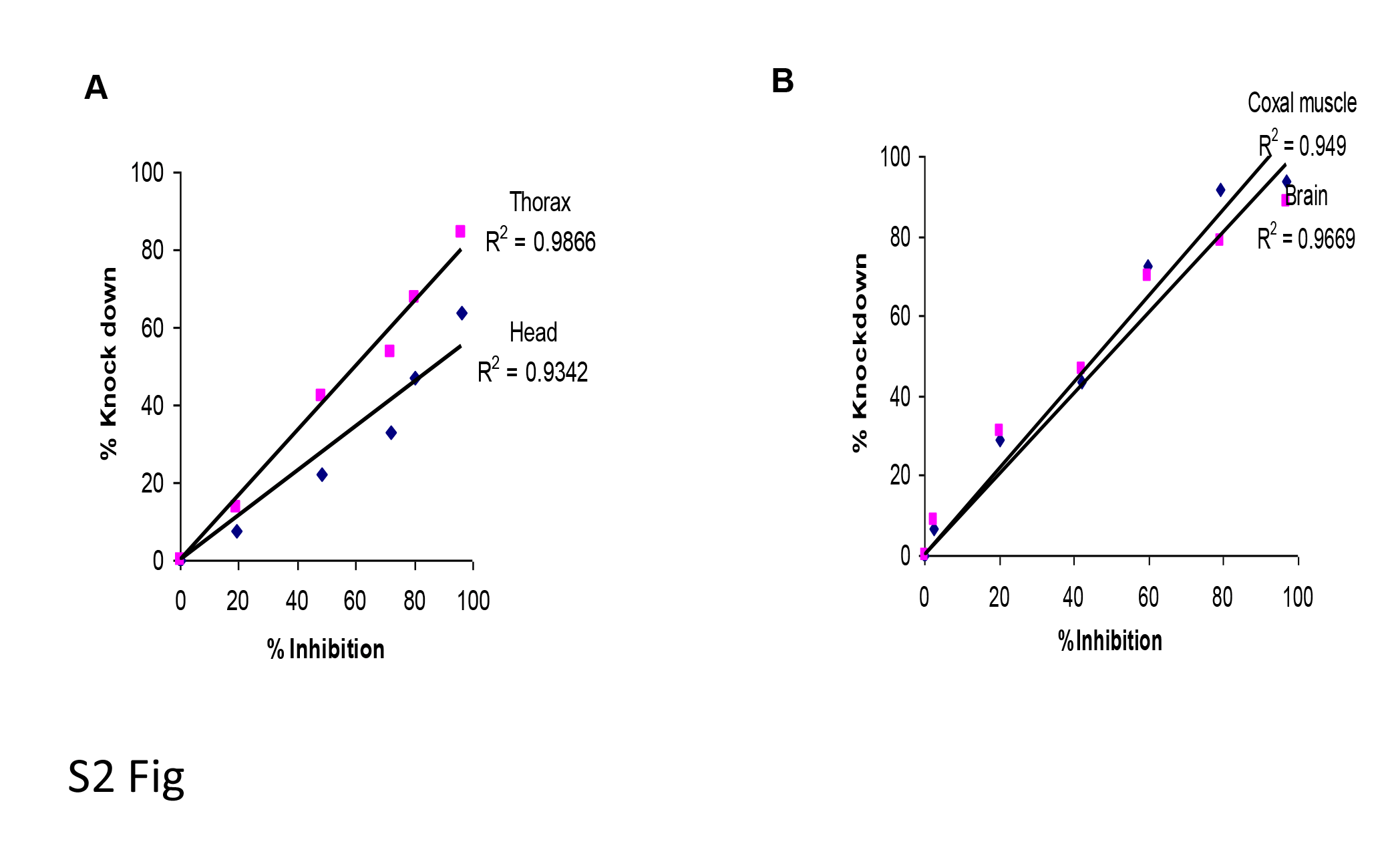

Supplement: S2 Fig — (TIF) [file pone.0170836.s002.tif]

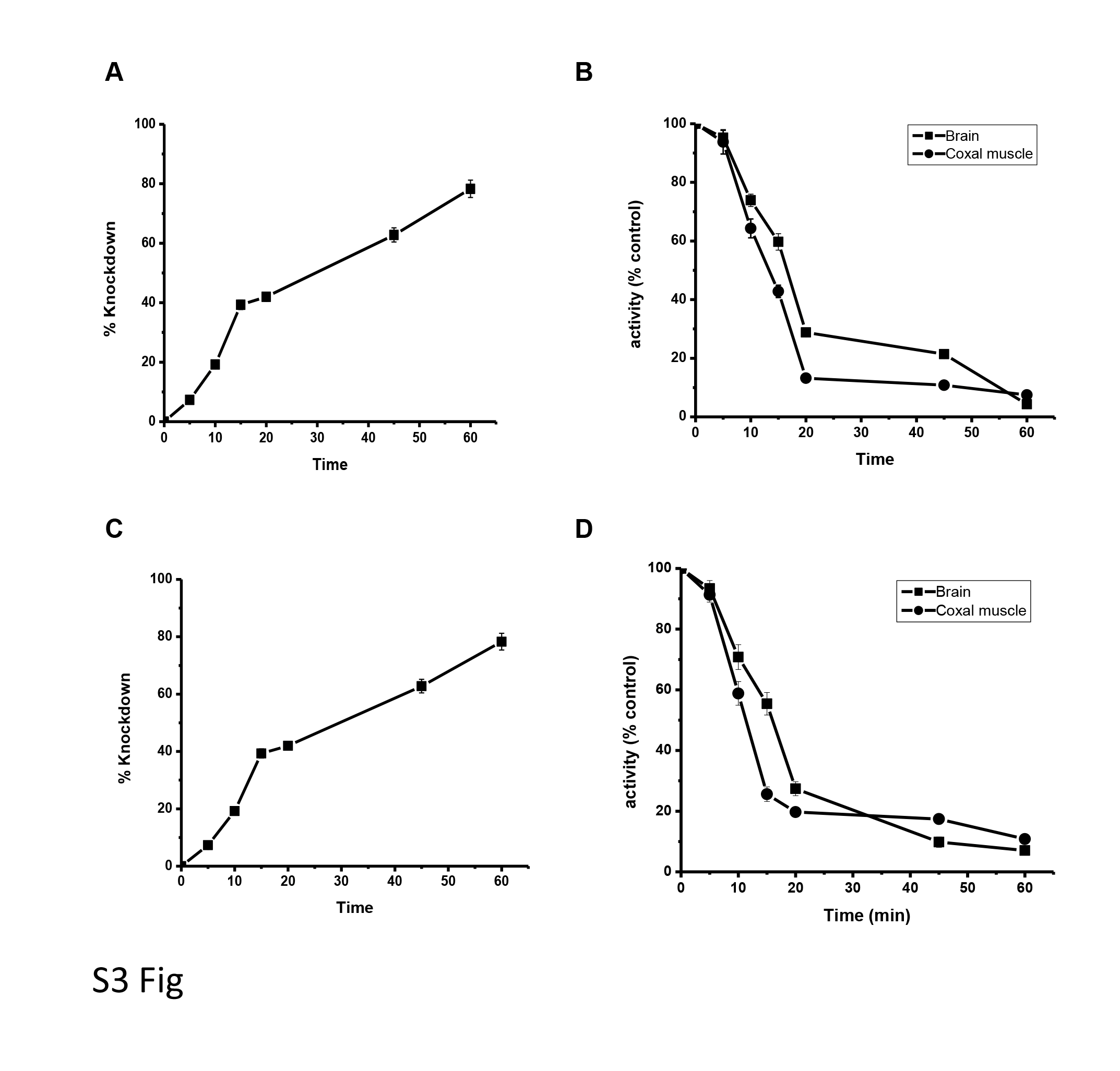

Supplement: S3 Fig — A) % Knockdown, B) Na+, K+ ATPase activity of decaleside II in Blatella germanica exposure at 1mg/leg (fore legs) by topical application (control activity: brain = 68.03 μg Pi / mg protein; coxal muscle = 75.8 μg Pi / mg protein). C) % Knockdown, D) Na+, K+ ATPase activity of decaleside II in Blatella germanica exposure at KD50 (0.07 mg/cm2) by contact bioassay (control activity: brain = 73.4.03 μg Pi / mg protein; coxal muscle = 78.8 μg Pi / mg protein). (TIF) [file pone.0170836.s003.tif]

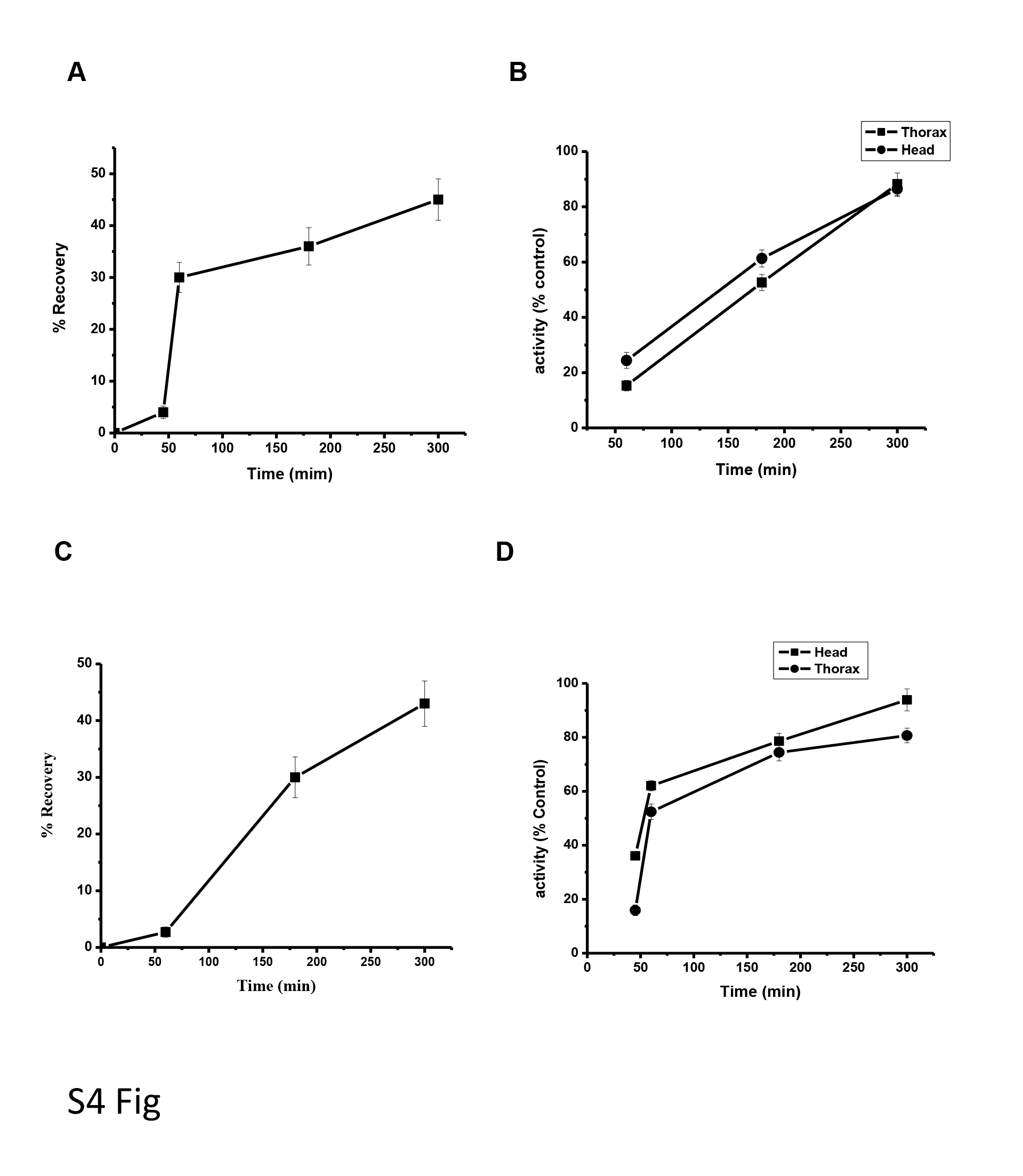

Supplement: S4 Fig — A) Recovery from knockdown of decaleside I; B) Recovery of Na+, K+-ATPase inhibition (control activity: head = 37.56 μg Pi / mg protein; thorax = 17.4 μg Pi / mg protein). C) Recovery from knockdown of decaleside II; D) Recovery of Na+, K+-ATPase inhibition (control activity: head = 37.56 μg Pi / mg protein; thorax = 17.4 μg Pi / mg protein). (TIF) [file pone.0170836.s004.tif]

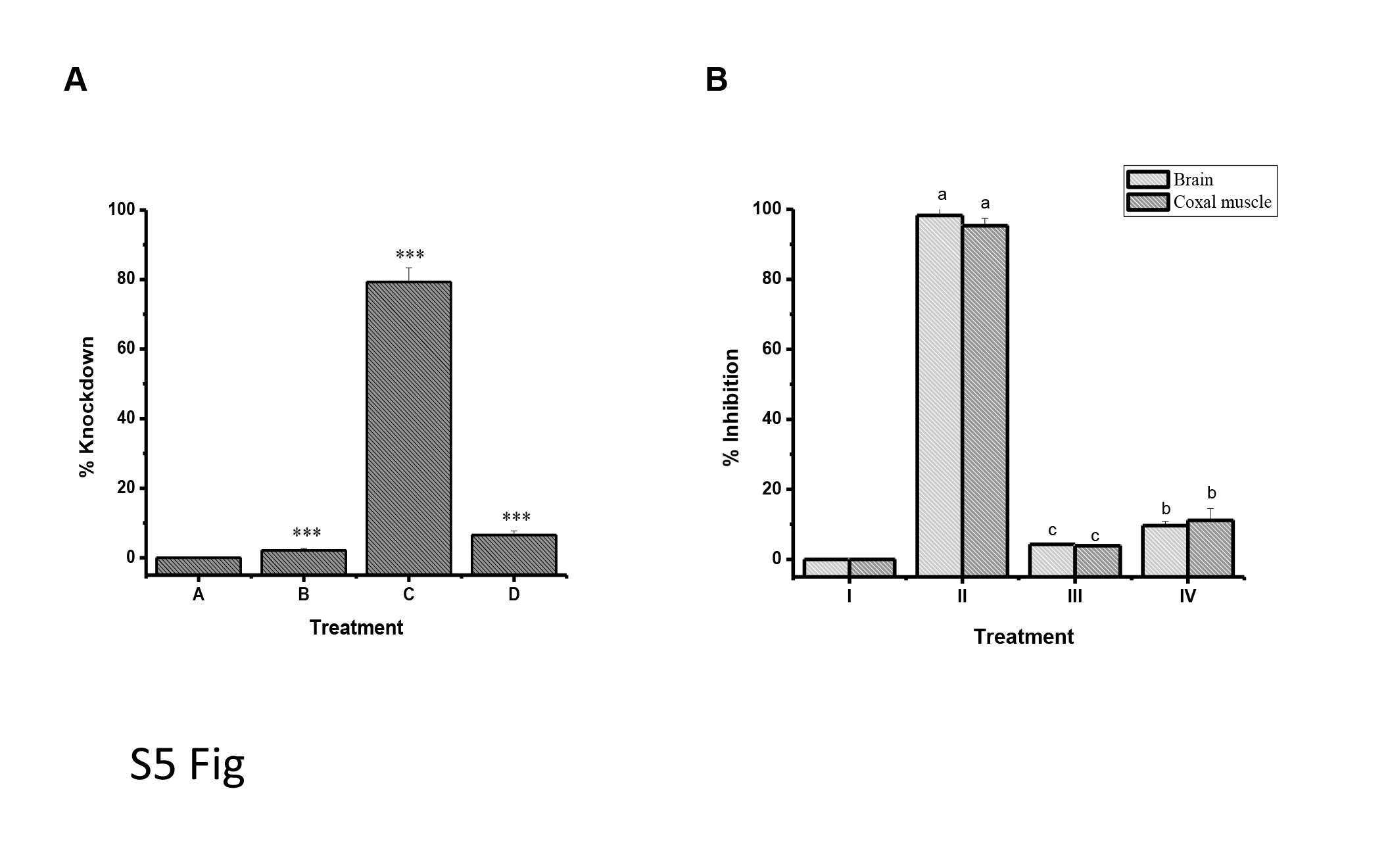

Supplement: S5 Fig — I) Control (no wax), II) Control (no wax) + decaleside II (1mg/insect), III) wax treated on tarsi (+ solvent), IV) with wax treated tarsi + decaleside II (1mg/insect) (n = 4, error bars, s.e.m.), One-way ANOVA, ***P < 0.001. (TIF) [file pone.0170836.s005.tif]

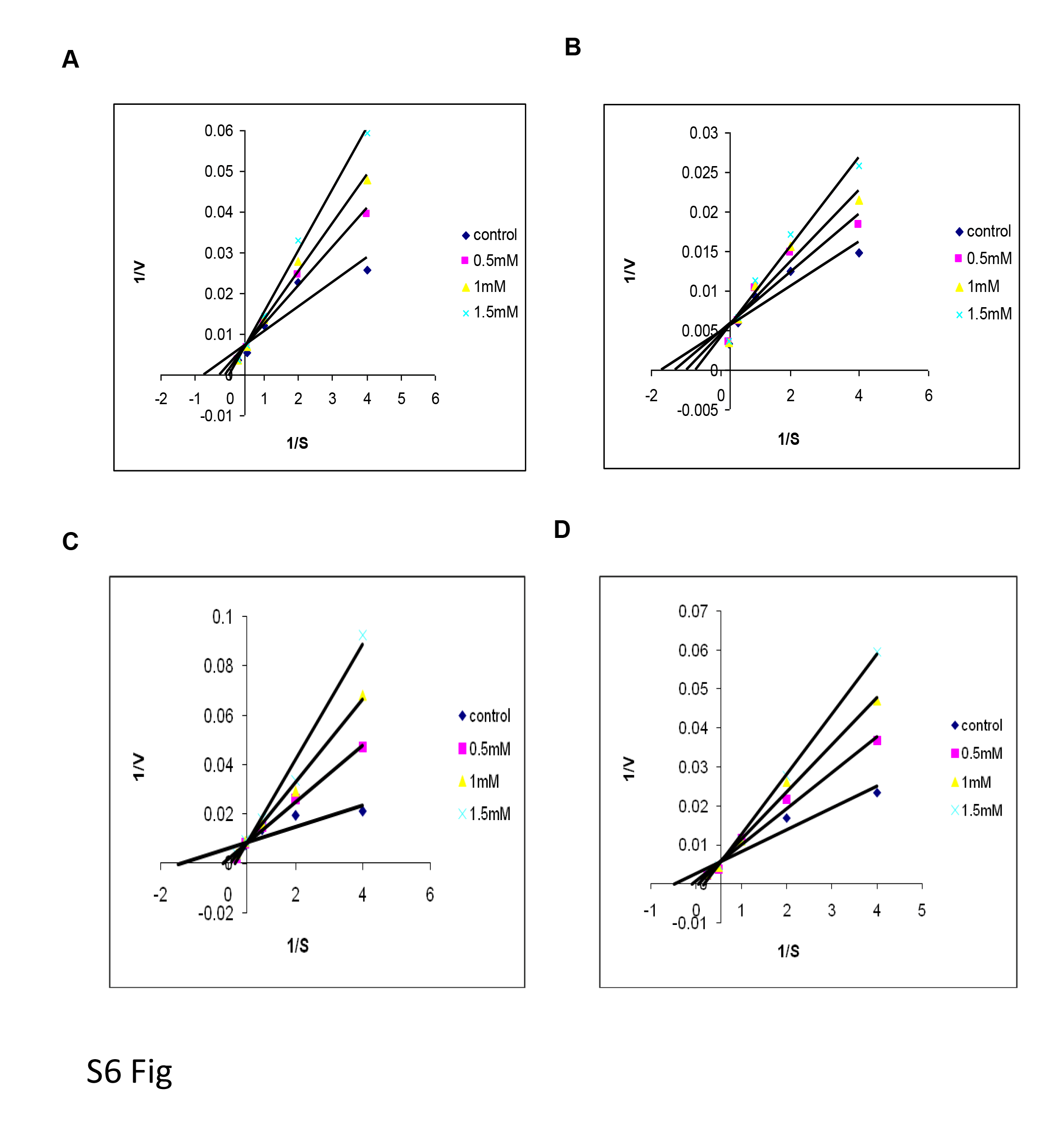

Supplement: S6 Fig — Brain: A, Decaleside I; B, Decaleside II. Coxal muscle: C, Decaleside I; D, Decaleside II. (TIF) [file pone.0170836.s006.tif]

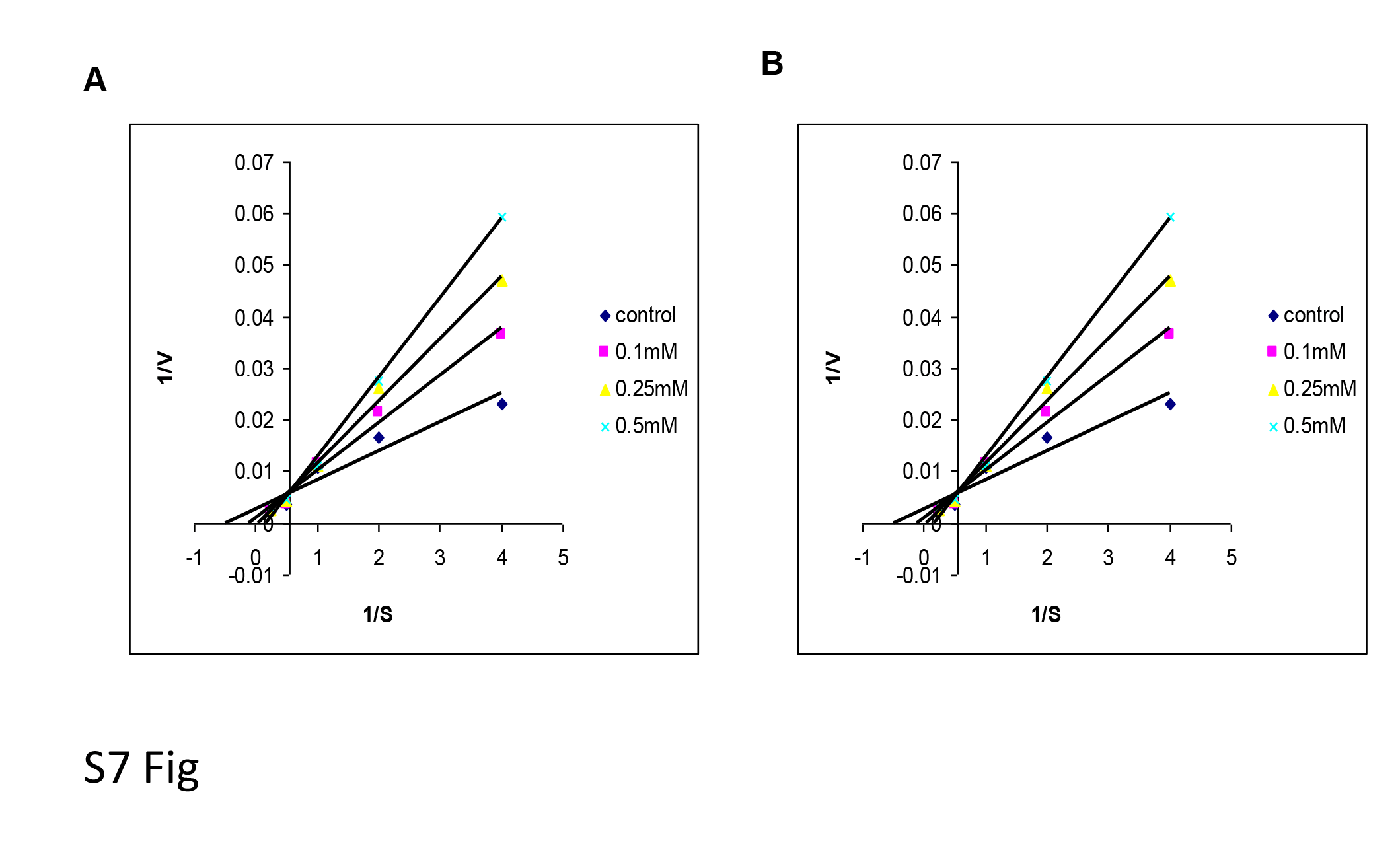

Supplement: S7 Fig — A) Decaleside I; B) Decaleside II. (TIF) [file pone.0170836.s007.tif]
