# Supplementary material for: Mode of Action of the Natural Insecticide, Decaleside Involves Sodium Pump Inhibition
Source: PLoS One. 2017 Jan 26;12(1):e0170836. doi: 10.1371/journal.pone.0170836 (PMC5268410; doi:10.1371/journal.pone.0170836)
Supplement: S1 Table — (PDF) [file pone.0170836.s008.pdf]

S1 Table: In vitro inhibition (IC_{50}) of Na^+, K^+ ATPase by decaleside I and II in the house fly, cockroach and purified Na^+, K^+ ATPase (porcine cerebral cortex).

**Inhibitors**	**IC_{50} (10^{-5}M)** (n = 4, mean ± SE)				
	**House fly**		**Cockroach**		**purified Na^+, K^+ATPase (porcine cerebral cortex)**
	Head	Thorax	Brain	Coxal Muscle	
**Decaleside I**	27.0 ± 0.8	31.0 ± 0.3	23.4 ± 0.6	23.0 ± 1.8	10.5 ± 0.8
**Decaleside II**	14.6 ± 0.4	19.3 ± 0.2	15.2 ± 0.4	16.8 ± 1.2	9.50 ± 0.4
**Ouabain**	54.0 ± 1.2	94.0 ± 2.6	101± 3.8	98.0 ± 2.4	25.4 ± 1.8
